# Geospatial datasets in support of high-resolution spatial assessment of population vulnerability to climate change in Nepal

**DOI:** 10.1016/j.dib.2017.04.045

**Published:** 2017-04-29

**Authors:** Janardan Mainali, Narcisa G. Pricope

**Affiliations:** a Department of Earth and Ocean Science, University of North Carolina Wilmington, 601 South College Road, Wilmington, NC 28403-5944, United States; b Research and Development Society, Nepal

## Abstract

We present a geographic information system (GIS) dataset with a nominal spatial resolution of one-kilometer composed of grid polygons originally derived and utilized in a high-resolution climate vulnerability model for Nepal. The different data sets described and shared in this article are processed and tailored to the specific objectives of our research paper entitled “High-resolution Spatial Assessment of Population Vulnerability to Climate Change in Nepal” (Mainali and Pricope, In press) [1]. We share these data recognizing that there is a significant gap in regards to data availability, the spatial patterns of different biophysical and socioeconomic variables, and the overall population vulnerability to climatic variability and disasters in Nepal. Individual variables, as well as the entire set presented in this dataset, can be used to better understand the spatial pattern of different physical, biological, climatic, and vulnerability characteristics in Nepal. The datasets presented in this article are sourced from different national and global databases and have been statistically treated to meet the needs of the article. The data are in GIS-ready ESRI shapefile file format of one-kilometer grid polygon with various fields (columns) for each dataset.

**Specifications Table**

**Table:** 

Subject area	Geography
More specific subject area	Climate vulnerability
Type of data	Geographic information system shape file
How data was acquired	From various secondary sources
Data format	Different levels of analysis
Experimental factors	Different data sets are normalized
Experimental features	Very brief experimental description
Data source location	Nepal
Data accessibility	Data is submitted with the article
Related research article	[1]

**Value of the data**

• It is a first one-kilometer resolution information of various biophysical and socio-economic data for Nepal used to derive population vulnerability to climate change and variability.
• It can be used by various organizations, local governments, and other researchers as a starting point to understanding climate vulnerability of Nepal.
• These data can be used to calculate various indices or components of vulnerability such as exposure, sensitivity, adaptive capacity, physiography, and socio-economic characteristics from a village scale to National scale in Nepal.
• The data and organization of this dataset can serve as a methodological transferability tool to help organize similar analyses in other locales.

## 1 Data

The data we are publishing here are processed information we created for the high-resolution climate vulnerability analysis in Nepal. The data is about one-kilometer resolution polygon shape file. These data are sourced from various national and global database as referenced in our original article [1]. Due credit has been given to all the sources we obtained the original data from. The data quantifying various biophysical and socioeconomic characteristics were used to derive climate vulnerability of Nepal. The individual datasets are presented as a column in an attribute table of the shape file.

## 2 Experimental design, materials and methods

In this dataset, we present a shapefile with one-kilometer grid (~0.0083°) for the country of Nepal and include 36 different variables we created (Table 1). Among them, 13 variables are created from different secondary databases from various sources. These 13 variables underwent different statistical treatments so as to derive the rest of the variables. Please refer to our article [1] for the data sources and detailed procedures of data processing. We based part of our methodology to create these variables on the approach employed by de Sherbinin et al. [2].

**Table 1:** Name and description of variables available in shapefile (MainaliPricopeData.shp).

**SN**	**Variable name in shapefile**	**Variable description**
1	prcp	Average precipitation (mm)
2	prcp_cov	Coefficient of variation of Precipitation (mm)
3	temp_trend	Temperature trend (°C/yr)
4	ndvi_std	Standard deviation of NDVI
5	slope	Slope (deg)
6	floodFreq	Flood frequency (Number per 100 years)
7	soc	Soil organic carbon (gm per thousand grams of soil)
8	landCover	Land cover (Rank)
9	IrrigL	Irrigation (Percentage)
10	wealth_ind	Household wealth Index (Rank)
11	femaleHH	Percentage of households with female head (Percentage)
12	healthInfr	Health Infrastructure (Rank)
13	distanceCi	Distance to city (min)
14	temp_std	Standardized variable of temperature trend
15	prcp_std	Standardized variable of average precipitation
16	prcp_cov_s	Standardized variable of coefficient of variation of precipitation
17	ndvi_std_s	Standardized variable of standard deviation of NDVI
18	slope_std	Standardized variable of slope
19	flood_std	Standardized variable of flood frequency
20	soc_std_1	Standardized and inverted variable of soil organic carbon
21	landC_std	Standardized variable of land cover rank
22	irrig_std_	Standardized and inverted variable of percentage of irrigated land
23	wealth_std_1	Standardized and inverted variable of household wealth index
24	female_std	Standardized variable of percentage of households with female head
25	health_std_1	Standardized and inverted variable of Health Infrastructure
26	distance_s	Standardized variable of distance to city
27	exposure_std	Standardized variable of exposure index
28	sensitivit	Standardized variable of sensitivity index
29	lackA_std	Standardized variable of lack of adaptive capacity index
30	averageVuln	Standardized variable of additive climate vulnerability index
31	physiograp	Physiography region
32	pc1_std_1	Standardized and inverted variable of loading in first principle component
33	pc2_std	Standardized variable of loading in second principle component
34	pc3_std	Standardized variable of loading in third principle component
35	pc4_std	Standardized variable of loading in fourth principle component
36	vuln_pc124_Std	Standardized variable of principal component-based vulnerability index
